# Accuracy of cuff blood pressure and systolic blood pressure amplification

**DOI:** 10.1038/s41440-023-01311-0

**Published:** 2023-05-22

**Authors:** Tan V. Bui, Dean S. Picone, Martin G. Schultz, Xiaoqing Peng, J. Andrew Black, Nathan Dwyer, Philip Roberts-Thomson, Heath Adams, Chen-Huan Chen, Hao-min Cheng, Giacomo Pucci, Jiguang Wang, Remi Goupil, James E. Sharman

**Affiliations:** 1 Menzies Institute for Medical Research, University of Tasmania, Hobart, TAS Australia; 2 Department of Obstetrics and Gynecology, The First Affiliated Hospital of Anhui Medical University, Hefei, China; 3 Royal Hobart Hospital, Hobart, TAS Australia; 4 Department of Medicine, National Yang Ming Chiao Tung University, Department of Medical Education, Taipei Veterans General Hospital, Taipei, Taiwan; 5 Unit of Internal Medicine at Terni University Hospital, Department of Medicine, University of Perugia, Perugia, Italy; 6 Centre for Epidemiological Studies and Clinical Trials, Shanghai Key Laboratory of Hypertension, The Shanghai Institute of Hypertension, Department of Hypertension, Ruijin Hospital, Shanghai Jiao Tong University School of Medicine, Shanghai, China; 7 Hopital du Sacre-Coeur de Montreal, Universite de Montreal, Montreal, Canada

**Keywords:** Sphygmomanometers, blood pressure determination, angiography, pulse wave analysis

## Abstract

Automated cuff measured blood pressure (BP) is the global standard used for diagnosing hypertension, but there are concerns regarding the accuracy of the method. Individual variability in systolic BP (SBP) amplification from central (aorta) to peripheral (brachial) arteries could be related to the accuracy of cuff BP, but this has never been determined and was the aim of this study. Automated cuff BP and invasive brachial BP were recorded in 795 participants (74% male, aged 64 ± 11 years) receiving coronary angiography at five independent research sites (using seven different automated cuff BP devices). SBP amplification was recorded invasively by catheter and defined as brachial SBP minus aortic SBP. Compared with invasive brachial SBP, cuff SBP was significantly underestimated (130 ± 18 mmHg vs. 138 ± 22 mmHg, *p* < 0.001). The level of SBP amplification varied significantly among individuals (mean ± SD, 7.3 ± 9.1 mmHg) and was similar to level of difference between cuff and invasive brachial SBP (mean difference –7.6 ± 11.9 mmHg). SBP amplification explained most of the variance in accuracy of cuff SBP (R^2^ = 19%). The accuracy of cuff SBP was greatest among participants with the lowest SBP amplification (p_trend_ < 0.001). After cuff BP values were corrected for SBP amplification, there was a significant improvement in the mean difference from the intra-arterial standard (*p* < 0.0001) and in the accuracy of hypertension classification according to 2017 ACC/AHA guideline thresholds (*p* = 0.005). The level of SBP amplification is a critical factor associated with the accuracy of conventional automated cuff measured BP.

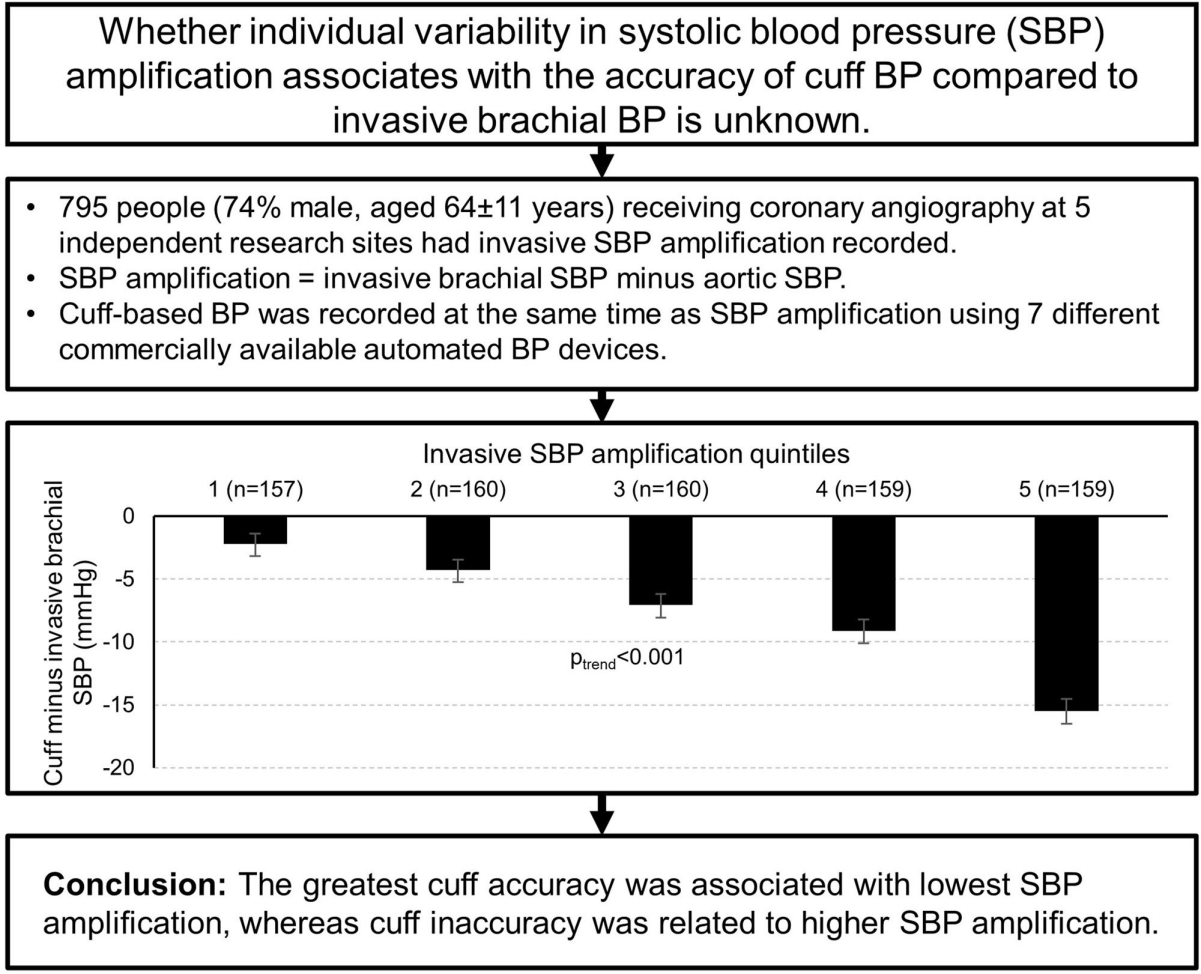

## Introduction

High blood pressure (BP) is the leading modifiable risk factor for cardiovascular disease and contributes to more than 10 million deaths annually [1]. Accurate BP measurement is a critical component of the healthcare pathway to enable correct identification of high BP, and consequent BP management to reduce risk for cardiovascular disease events [2]. International guidelines recommend that appropriately validated, upper arm cuff-based, automated BP measuring devices are used for clinical diagnosis and management [3]. Irrespective of the clinical value of automated cuff BP devices, accuracy concerns that could influence correct diagnosis in some people have been raised, specifically that the cuff BP does not accurately represent the true intra-arterial BP values [4]. Indeed, cuff systolic BP (SBP) systematically underestimates intra-arterial brachial SBP, whereas cuff diastolic BP (DBP) systematically overestimates intra-arterial brachial DBP (both by about 6 mmHg on average) [5]. Better understanding of the mechanisms underlying the inaccuracy of cuff BP could help towards refining accuracy and improving individual BP risk stratification.

The operating principles of standard automated (oscillometric) cuff devices are based on analysis of pressure (or volume) waveforms detected at the brachial artery. These waveforms are extracted from the cuff pressure (deflation) curve and processed to construct an oscillometric waveform envelope [6]. From this, the mean arterial pressure is identified from the maximal amplitude, and proprietary algorithms are employed to estimate SBP and DBP [7]. These brachial artery waveforms captured by cuff devices have characteristic morphology that are unique to each individual [8] but to our knowledge they are not considered in the proprietary algorithms used to estimate BP parameters. Recently we found that the arterial waveform morphology at the brachial artery was distinct among people with high level of central to brachial SBP amplification (e.g. >15 mmHg; relatively high amplitude, narrow systolic peak) compared to those with low SBP amplification (lower amplitude, broad systolic wave) [9]. These characteristic differences in waveform morphology could result in systematic bias in automated cuff BP measurement that is dependent on SBP amplification. If this were the case, we may expect to see a positive association between the magnitude of SBP amplification and the magnitude of error in cuff measured BP. The aim of this study was to determine the relationship between individual variability in SBP amplification and the accuracy of cuff BP compared with intra-arterial brachial BP. Given that different cuff BP devices have unique proprietary algorithms to estimate BP, we wanted to confirm findings in a large subject population across several models of cuff BP devices.

## Methods

### Study population

Participants were 795 patients undergoing coronary angiography who were recruited from five independent research sites using a variety of automated cuff BP devices. At each study site clinical characteristics, cuff BP, invasive brachial BP and invasive aortic BP measurements were recorded in accordance with available international standard guidelines [10] and then combined as a convenience sample. Interarm differences in cuff BP were recorded as a screening measure for eligibility, and only those subjects with no major interarm BP difference proceeded to the invasive measurements. This was as a quality control measure to rule out those with possible upper limb stenosis or hemodynamic abnormality that could influence accurate BP measurements. Further details on the study population, inclusion and exclusion criteria are provided in published studies [11–15] from the different research sites and are summarised in Supplementary Table 1. The current analysis includes data but is completely separate from our previous published paper [15]. Participants provided written, informed consent at each research site.

### Non-invasive (cuff-based) and invasive brachial BP difference

Cuff brachial BP was recorded using seven different commercially available automated cuff BP devices (Supplementary Table 1). Cuff BP was measured precisely simultaneous to invasive brachial BP in five studies (four published [11, 14, 15], one unpublished), and immediately prior to invasive brachial BP in two studies [12, 13]. Participants were excluded if there was an interarm difference >3 mmHg (in two published studies [11, 14]) or >5 mmHg (in four published studies [12, 13, 15], one unpublished). In all studies, only those subjects without significant interarm differences went on to have cuff BP and invasive BP recorded on opposite arms. A total of 166 participants were excluded on the basis of interarm BP differences. Non-invasive (cuff) SBP, DBP and pulse pressure (PP) difference (inaccuracy) was calculated as cuff minus invasive brachial SBP, DBP and PP.

### Invasive (intra-arterial) SBP amplification

Details of intra-arterial BP collection procedures for each individual study are provided in previous publications [11–15] and are summarised in Supplementary Table 1. Briefly, a solid-state or fluid-filled catheter was advanced from the right radial artery access site and positioned in the ascending aorta within 1–5 cm of the aortic valve, with confirmation by fluoroscopy. In six of the seven studies [11–13, 15], intra-arterial BP was measured by positioning a catheter in the ascending aorta to capture invasive aortic BP waveforms and then pulled back to the mid-humeral level in the right brachial artery to record invasive brachial BP waveforms. In one study, a dual-sensor, solid-state catheter allowed simultaneous measurement of invasive aortic and brachial BP [14]. Invasive SBP, DBP and PP amplification were defined as invasive brachial minus invasive aortic SBP, DBP and PP.

### Statistical analysis

Clinical characteristics and BP are presented as mean ± SD or *n* (%). Differences between continuous clinical characteristics and BP measures were assessed by *t* tests or one-way ANOVA with post hoc Tukey HSD test to quantify the statistical significance of any differences. Agreement between cuff and invasive brachial SBP was assessed by mean difference and SD of the mean difference and visualised by Bland Altman plots [16]. Pearson correlation and linear regression within Bland-Altman plots were used to determine the magnitude and direction of any proportional systematic bias (comparing correlation coefficients using Fisher’s z). The association between cuff and invasive brachial BP difference and invasive SBP amplification was assessed using univariable and multivariable linear regression adjusting for potential confounders including sex, age, height, coronary artery disease, heart rate, and mean invasive brachial arterial pressure. These variables were included in the adjusted models because they were associated or had suspected associations with the difference and invasive SBP amplification. An analysis for the use of antihypertensive medication was also conducted on data from a subgroup of participants with available data. The total number of participants was 795 for all analyses except the multiple regression, in which complete data was available for 755 participants (Supplementary Fig. 1). Since the average level of cuff SBP underestimation was similar to the average level of SBP amplification, we performed a cuff SBP correction to determine if this resulted in an improvement in the mean difference of cuff SBP from the invasive standard. The cuff SBP correction was performed by adding each individual’s level of invasive SBP amplification to their cuff SBP measure. BP stages were classified using brachial cuff BP, corrected brachial cuff BP and invasive brachial BP. Since brachial cuff measurements used to classify hypertension categories were corrected for invasive SBP amplification, invasive brachial BP was used as the reference standard. All classifications were according to the 2017 American College of Cardiology/American Heart Association (ACC/AHA) guidelines [3]. The concordance of BP classifications was assessed by comparing BP classification defined using cuff brachial BP with the one obtained using invasive brachial BP. Similar BP classification concordance was performed between corrected cuff BP and invasive brachial BP. These classification concordances were compared using kappa statistics, proportions of agreement and the two-proportion tests. Statistical analyses were performed using STATA version 17.0, *p* values ≤ 0.05 were considered statistically significant.

## Results

### Clinical characteristics

Clinical characteristics are outlined within Table 1 Participants were generally representative of patients undergoing coronary angiography, who were on average, of older age and higher body mass index, and more than half the population had hypertension based on cuff BP values according to the 2017 ACC/AHA guidelines. More than two thirds of participants reported taking at least one hypertensive medication.

**Table 1:** Participant characteristics and clinical measures (*n* = 795)

Participant characteristics	
Male sex, *n* (%)	585 (73.6)
Age (years)	64 ± 11
Height (cm)	168.5 ± 9.8
Weight (kg)	80.6 ± 18.0
Body mass index (kg/m^2^)	28.2 ± 5.0
Estimated glomerular filtration rate (ml/min/1.73m^2^)	79.6 ± 19.5
Coronary artery disease, *n* (%)	408 (53.3)
Type 2 diabetes mellitus, *n* (%)	206 (27.0)
Hypertension^a^, *n* (%)	
Cuff BP	447 (56.2)
Invasive brachial BP	506 (63.7)
Invasive aortic BP	400 (50.3)
Antihypertensive medications^b^, *n* (%)	569 (75.4)

Data are mean ± standard deviation or *n* (%);

*BP* blood pressure, *SBP* systolic BP, *DBP* diastolic BP;

^a^Hypertension was based on cuff, invasive brachial and aortic BP values and defined as SBP ≥ 130 mmHg or DBP ≥ 80 mmHg according to the 2017 ACC/AHA guidelines;

^b^Having at least one antihypertensive medication;

*n* varies due to missing data

### BP measurements

**Fig. 1:**
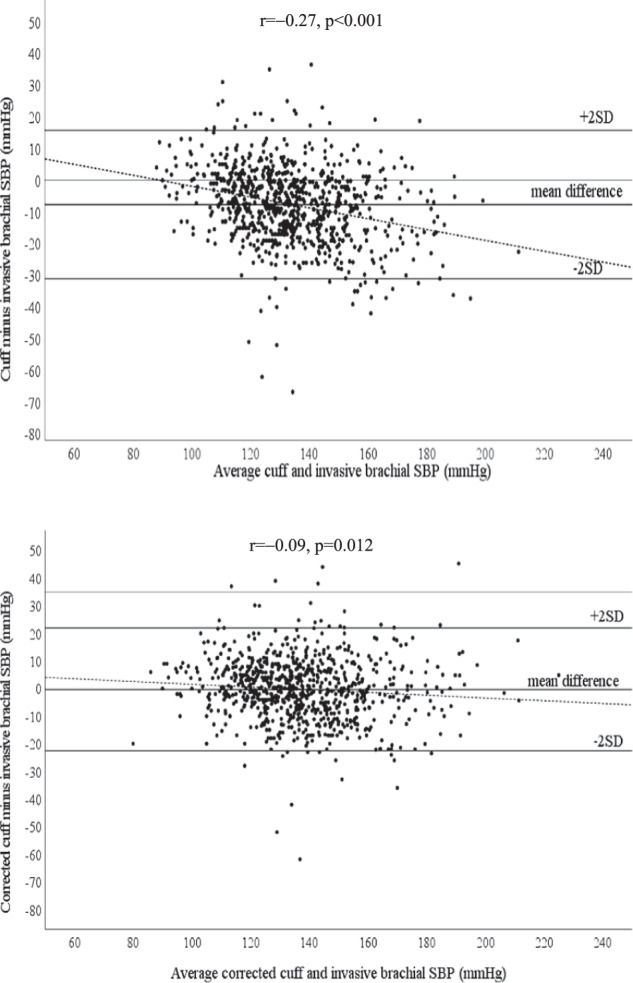
Bland-Altman plots of differences between cuff SBP (top) and cuff SBP corrected with SBP amplification (bottom) and invasive brachial SBP. Dashed lines represent the lines of best fit. Solid lines are mean difference ± 2 SDs. Bland-Altman plots show wide scatter and evidence of systematic bias for greater underestimation of invasive brachial SBP with increasing level of BP, but the slope of this association was significantly attenuated when cuff SBP was corrected by adding individual corresponding SBP amplification (*r* = −0.27 vs. *r* = −0.09, *z* = 5.25, *p* < 0.0001). There was a significant improvement in the mean difference between standard cuff and corrected cuff SBP from invasive brachial SBP (−7.6 ± 11.9 mmHg vs. −0.30 ± 11.4 mmHg, *p* < 0.001)

Cuff and invasive BP measurements are outlined within Table 2. Cuff-measured SBP significantly underestimated invasive brachial SBP (130 ± 18 mmHg vs. 138 ± 22 mmHg, *p* < 0.001), whereas cuff DBP significantly overestimated invasive brachial DBP (76 ± 11 mmHg vs. 69 ± 10 mmHg, *p* < 0.001). Bland-Altman plots revealed significant bias for cuff SBP to overestimate invasive brachial SBP at lower BP levels but underestimate invasive brachial SBP at higher BP levels (Fig. 1). The slope of the bias was significantly attenuated after correcting cuff SBP for the corresponding level of SBP amplification for each individual (*r* = −0.27 vs. *r* = −0.09, *z* = 5.25, *p* < 0.0001). There was also a significant improvement in the mean difference, but not standard deviation, between cuff SBP and invasive brachial SBP when cuff SBP was corrected with SBP amplification (−7.6 ± 11.9 mmHg vs. −0.3 ± 11.4 mmHg, *p* < 0.001). Although there was wide individual variability in SBP amplification (mean ± SD, 7.3 ± 9.1 mmHg), the mean SBP amplification was similar to the mean difference between cuff SBP and invasive brachial SBP (−7.6 ± 11.9 mmHg). Analysis of cuff and invasive BP measurement was also conducted for each of the seven devices across all the study sites (Supplementary Table 2). Findings were broadly similar with the pooled analysis except for the device used by Ding et al. [12], where an underestimation (instead of overestimation) of cuff DBP was observed.

**Table 2:** Participant cuff and invasive blood pressure measurements (*n* = 795)

Cuff BP (mmHg)	
SBP	130 ± 18
DBP	76 ± 11
PP	54 ± 14
MAP	94 ± 12
Heart rate (bpm)	67 ± 12
Invasive brachial BP (mmHg)	
SBP	138 ± 22
DBP	69 ± 10
PP	69 ± 19
MAP	92 ± 12
Invasive aortic BP (mmHg)	
SBP	130 ± 21
DBP	70 ± 10
MAP	90 ± 12
Invasive BP amplification (mmHg)^a^	
SBP	7.3 ± 9.1
DBP	−0.8 ± 4.4
PP	8.1 ± 8.6
Cuff SBP corrected with SBP amplification (mmHg)^b^	137 ± 21
Cuff BP differences (mmHg)^c^	
Cuff SBP – invasive brachial SBP	−7.6 ± 11.9
Cuff DBP – invasive brachial DBP	7.4 ± 8.3
Cuff PP – invasive brachial PP	−15.0 ± 12.6

Data are mean ± standard deviation; *BP* blood pressure, *SBP* systolic blood pressure, *DBP* diastolic blood pressure, *PP* pulse pressure, *MAP* mean arterial pressure

^a^Invasive BP amplification was defined as invasive brachial BP minus invasive aortic BP;

^b^Individual cuff SBP was added by each corresponding SBP amplification;

^c^Cuff BP differences were defined as cuff minus invasive brachial BP

### Relationship of BP accuracy with SBP amplification

**Fig. 2:**
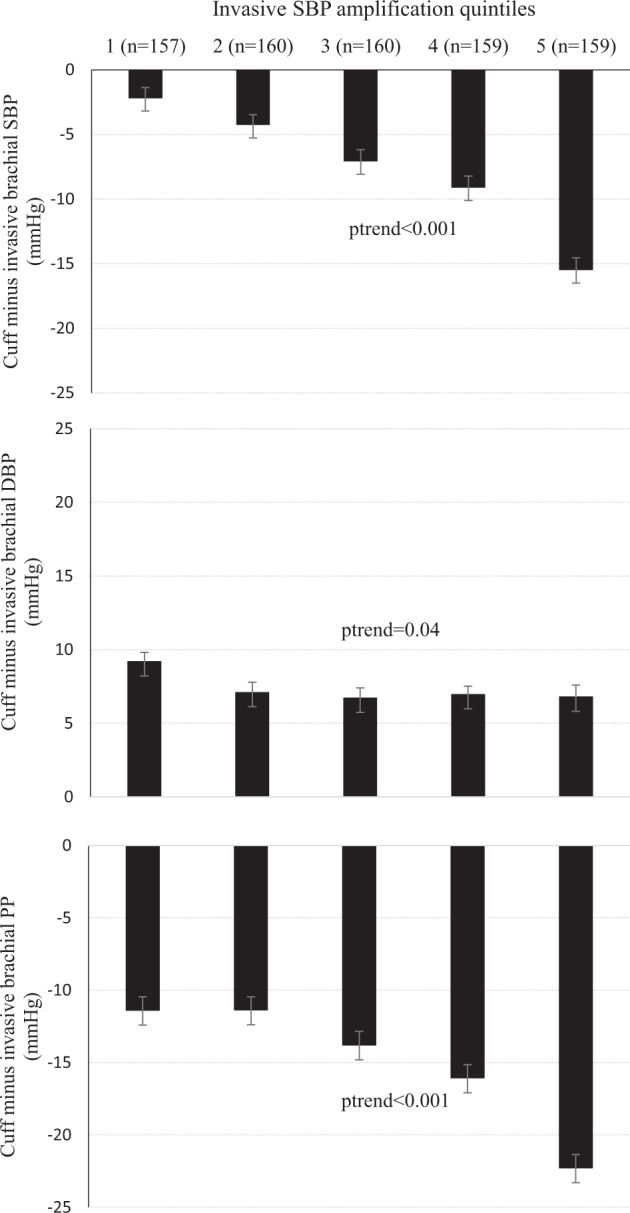
Bar plots (mean, SE) cuff minus invasive brachial systolic blood pressure (SBP, top), diastolic (DBP, middle) and pulse pressure (PP, bottom) by invasive SBP amplification quintiles. There was a stepwise increase in mean differences between cuff and invasive brachial SBP, PP for each of elevated invasive SBP quintile (p_trend_ < 0.001) whilst there was a slight decrease for DBP (*p* = 0.04)

Fig. 2 presents differences between cuff and invasive brachial SBP, DBP, and PP by invasive SBP amplification quintiles. There was a significant trend towards greater underestimation of cuff SBP across quintiles of SBP amplification (p_trend_ < 0.001). The accuracy of cuff SBP was greatest among participants with the lowest SBP amplification. The difference between cuff and invasive brachial SBP was significantly associated with SBP amplification, even after controlling for multiple potential confounders (β[95%CI]:–0.52[–0.60 to –0.44], *p* < 0.001) and SBP amplification explained most of the variance in accuracy of cuff SBP (R^2^ = 19%, Table 3). Participant characteristics, cuff, invasive brachial and aortic BP data used in the adjusted models were compared in Supplementary Table 3. There were no significant differences between the missing (*n* = 40) and the complete (*n* = 755) datasets for the proportion of male participants and coronary artery disease, mean age, invasive SBP amplification, cuff and invasive brachial difference (*p* > 0.065, all). Results were unchanged when adjusted for the use of antihypertensive medication (Supplementary Table 4). Supplementary Table 5 presents participants clinical characteristics and BP measures across quintiles of invasive SBP amplification. Age, height, coronary artery disease, heart rate, invasive brachial SBP, PP, mean arterial pressure, invasive aortic SBP, DBP and mean arterial pressure were significantly different across these quintiles (p_trend_ ≤ 0.023 for all).

**Table 3:** Association between cuff SBP, DBP and PP accuracy and invasive SBP amplification

	n^a^	β^b^	95%CI		R^2^(%)^c^	*p*
Cuff – invasive brachial SBP						
Unadjusted	755	−0.57	(−0.65 to −0.49)		18.8	<0.001
Adjusted^d^	755	−0.52	(−0.60 to −0.44)		32.7	<0.001
Cuff – invasive brachial DBP						
Unadjusted	755	−0.11	(−0.18 to −0.05)		1.4	0.001
Adjusted^d^	755	−0.08	(−0.14 to −0.01)		10.8	0.021
Cuff – invasive brachial PP						
Unadjusted	755	−0.46	(−0.55 to −0.36)		10.8	<0.001
Adjusted^d^	755	−0.45	(−0.53 to −0.36)		25.2	<0.001

*SBP* systolic blood pressure, *DBP* diastolic blood pressure, *PP* pulse pressure

^a^due to missing data, complete case analysis was conducted;

^b^unstandardised beta, invasive SBP amplification was defined as invasive brachial SBP minus invasive aortic SBP;

^c^adjusted R^2^;

^d^adjusted for sex, age, height, coronary artery disease, heart rate, and mean invasive brachial arterial pressure

### BP classification concordance

Concordance of 2017 ACC/AHA BP classification according to cuff and invasive brachial BP is presented in Table 4. Without correction for SBP amplification, there was fair agreement (Cohen κ, 0.42, and 57.4% concordance) between cuff and invasive brachial BP across BP classification thresholds. After cuff BP values were corrected for SBP amplification, there was an improvement in the accuracy of hypertension classification (Cohen κ, 0.49, and 63.7% concordance, *p* for two proportion test = 0.005). There were similar patterns of concordance when the 2020 International Society of Hypertension and 2018 European Society of Hypertension/European Society of Cardiology classification guidelines were applied (Supplementary Tables 6, 7 respectively).

**Table 4:** Concordance of BP classification according to cuff and invasive brachial BP^a^

Invasive brachial BP classification	Brachial cuff BP classification			
	Normal BP	Elevated BP	Stage 1 Hypertension	Stage 2 Hypertension
	*n*(%)	*n*(%)	*n*(%)	*n*(%)
Standard brachial cuff BP				
Normal BP	***125(55.1)***	19(15.7)	9(4.2)	2(0.9)
Elevated BP	59(26.0)	***38(31.4)***	31(14.4)	6(2.6)
Stage 1 Hypertension	28(12.3)	36(29.8)	***86(39.8)***	16(6.9)
Stage 2 Hypertension	15(6.6)	28(23.1)	90(41.7)	***207(89.6)***
(%) Agreement: 57.4%, kappa: 0.42, *p* < 0.001				
Cuff SBP corrected with SBP amplification^b^				
Normal BP	***101(74.3)***	36(30.3)	16(7.9)	2(0.6)
Elevated BP	21(15.4)	***52(43.7)***	43(21.3)	18(5.3)
Stage 1 Hypertension	11(8.1)	22(18.5)	***84(41.6)***	49(14.5)
Stage 2 Hypertension	3(2.2)	9(7.6)	59(29.2)	***269(79.6)***
(%) Agreement: 63.7%, kappa: 0.49, *p* < 0.001				

*P* value for the test comparing two proportions of concordance = 0.005;

*BP* blood pressure, *SBP* systolic blood pressure, *DBP* diastolic blood pressure

^a^Hypertension classification based on 2017 ACC/AHA guidelines: Normal BP: SBP < 120 mmHg and DBP < 80 mmHg; Elevated BP: SBP 120–129 mmHg and DBP < 80 mmHg; Stage 1 Hypertension: SBP 130–139 mmHg or DBP 80–89 mmHg; Stage 2 Hypertension: SBP ≥ 140 mmHg or DBP ≥ 90 mmHg;

Each column adds to 100%;

*n* (%) represent the number and percentage of concordance for each classification, bolded and italic numbers represent concordant classifications;

^b^Individual SBP amplification value was added to each corresponding cuff SBP value before applying ACC/AHA classification

## Discussion

The key novel finding of this study was that the individual level of SBP amplification was significantly associated with the accuracy of cuff measured SBP. This was confirmed in independent study samples and across several different automated cuff BP measurement devices, each using unique proprietary algorithms to estimate BP. The accuracy of cuff measured SBP was highest among individuals with the lowest levels of SBP amplification, and cuff SBP progressively underestimated intra-arterial brachial SBP as SBP amplification increased. Multiple regression analysis identified SBP amplification as the factor explaining most of the variance in the accuracy of cuff measured SBP compared with intra-arterial brachial SBP. To our knowledge, this is the first study to report such findings, which provide insight on a key factor relevant the accuracy of conventional upper arm automated cuff BP methods.

The observed relationship between cuff accuracy and SBP amplification could be explained by the automated cuff measurement method itself, which has similar operating principles between devices (albeit having different algorithms) and employs analytical processes that are largely unchanged for decades [17–19]. A variety of propriety algorithms can be applied (e.g. using fixed-ratio coefficients) to estimate SBP and DBP at specific fractions of the envelope peak. These BPs are designed to copy the BP values recorded by manual auscultation [20, 21]. A critical factor relating to automated cuff BP compared with auscultatory BP is that individual differences in the shape of the waveform envelope result in different levels of accuracy of the estimated BP values [22–24]. Alongside this, we have observed phenotypic differences in the brachial arterial waveform shapes [9] (and consequent estimation of mean arterial pressure) [25] between individuals with low- compared to high- SBP amplification. It is possible these phenotypic waveform differences associated with SBP amplification, are influencing cuff accuracy via variability in the waveform envelope shape and consequent error in cuff SBP estimations. Such a problem could be rectified through development of BP estimation algorithms that are individualised based on pressure waveform characteristics associated with SBP amplification [26]. However, to date there are no mechanistic studies to determine whether the waveform envelope is influenced by BP amplification and arterial waveform shapes. Other factors potentially influencing the findings could include such things as local tissue properties under the cuff, the location of the cuff on the upper arm and cuff inflation or deflation rates.

This study identified a systematic error in the accuracy of cuff BP (compared with intra-arterial brachial BP) associated with SBP amplification and this has direct implications for accurate assessment of the true risk related to BP (the actual intra-arterial BP values). With respect to non-invasive cuff BP measurement, even small BP errors at the individual level can have large consequences on correct hypertension classification, prevalence and control [27]. An underestimation of 4/2 (SBP/DBP) mmHg corresponds to lowering hypertension prevalence but increasing hypertension control estimates by more than 5% respectively [28]. A small lowering in SBP (e.g. 2 mmHg) also correlates to about 7 to 10% reduction in ischemic heart disease and stroke mortality [29]. Given the consistency of our principal findings across seven separate automated BP measurement devices, the results may be applied to advance the individual level accuracy of standard cuff BP measurement compared with intra-arterial values, and thus achieve greater precision in cardiovascular risk stratification and treatment. However, the relative clinical value of invasive BP at either the central aorta or brachial artery has yet to be determined in large scale datasets, and cuff BP remains the clinical standard. As a point of interest, cuff SBP was the same average value as intra-arterial central aortic SBP, and explains the often-reported lack of SBP amplification between invasive central SBP and cuff SBP.

There are several study strengths, including a large population sample with high-quality intra-arterial measured brachial BP as the reference standard to confirm BP accuracy, as well as intra-arterial measurement of SBP amplification. Additionally, the findings were confirmed across five independent research sites, using seven independent automated BP devices. Potential limitations include a relatively homogenous clinical study sample comprising people with an indication for coronary angiography who are mostly older men and with multiple risk factors for cardiovascular disease. The results may therefore have limited generalisability beyond those with similar characteristics, although collecting invasive BP from healthy individuals without indication for coronary angiography is unethical. Although data were recorded according to guidelines [10], this is a convenience sample from several study centres and data collection protocols were not standardized across the study sites. The dynamic response of the fluid-filled catheter systems used to record pressure waveforms was assessed and confirmed to be in an appropriate range at four of the five study sites [12, 13, 15]. The findings from the study site that did not assess the dynamic response were in accordance with the pooled results from all studies. Effort was made to maintain the transducer at heart level throughout the research procedure at each site using fluid-filled catheters. However, a standardized protocol was not used to identify the phlebostatic axis and this could have led to hydrostatic errors. As an observational study, unmeasured confounding cannot be ruled out and causal inference of SBP amplification on cuff BP accuracy would need to be confirmed in an experimental design. Although correcting cuff SBP with SBP amplification improved the mean difference between cuff and invasive brachial SBP, the standard deviation remained similar, indicating no improvement in the precision (variance) of BP measurements after correction. This study is unable to determine the origin of the lack of change in variance, for example whether it is an intrinsic measurement issue or the added variance of the distribution of BP in the cohort. This question will need to be resolved to improve BP measurement precision within individuals. Finally, the accuracy of cuff BP was compared with invasive brachial BP because both measures are recorded at the same arterial site and cuff BP is the clinical standard. However, invasive central aortic BP may have stronger concordance with clinical outcomes than invasive brachial BP and cuff BP, but this needs to be determined in future studies.

In conclusion, this study found a significant association between the accuracy of conventional cuff measured BP (as it pertains to invasive brachial BP) and the magnitude of SBP amplification between the aorta and the site of cuff measurement – the brachial artery. Greatest cuff accuracy was associated with lowest SBP amplification, whereas cuff inaccuracy was related to higher SBP amplification. Enhancing the accuracy of BP measurement in clinical practice and research is an urgent and ongoing priority for major organisations worldwide [30, 31], and the findings from this study may ultimately be applied towards this goal.

## Supplementary information

Supplementary Tables and Figures
