# Supplementary material for: Accuracy of cuff blood pressure and systolic blood pressure amplification
Source: Hypertens Res. 2023 May 22;46(8):1961–9. doi: 10.1038/s41440-023-01311-0 (PMC10404511; doi:10.1038/s41440-023-01311-0)
Supplement: Supplementary file 1 — Supplementary Tables and Figures [file 41440_2023_1311_MOESM1_ESM.docx]

**Supplementary tables and figures**

| Subjects within cuff and invasive brachial blood pressure measurements  (n=795) | Excluded |
| --- | --- |
|  |  |
|  | Height  (n=39) |
|  |  |
|  | Heart rate  (n=1) |
|  |  |
| Total for complete case analysis  (n=755) |  |

Supplementary Figure 1. Participant flow showing exclusions from the complete case analysis.

| Supplementary Table 1. Participant characteristics, non-invasive and invasive BP measurements | | | | | | | | | |
| --- | --- | --- | --- | --- | --- | --- | --- | --- | --- |
| Study | n* | Age  (years)† | | Male  (%) | Brachial cuff method, device name | Catheter types, intra-arterial measurement method | Amplification measurement method | Cuff BP measurement protocol | Study exclusion criteria |
| Cheng et al, 2010 [11] | 115 | 63 | ±13 | 73.0 | Oscillometric, Omron VP-2000 device | Micromanometer tip | Catheter pulled back | Simultaneous with both invasive aortic and brachial BP | Acute coronary syndrome, peripheral arterial disease, abnormal sinus rhythm and >3mmHg pressure difference between left and right arms |
| Lin et al, 2012 [14] | 77 | 65 | ±13 | 79.2 | Oscillometric, Microlife WatchBP | Micromanometer tip | Simultaneous | Simultaneous with both invasive aortic and brachial BP | Acute coronary syndrome, peripheral arterial disease, abnormal sinus rhythm, or left/right arm BP difference >3mmHg pressure |
| Ding et al, 2013 [12] | 33 | 60 | ±9 | 63.6 | Oscillometric, HEM9000AI Omron | Fluid-filled | Catheter pulled back | Sequential cuff BP first then brachial BP within one to two minutes | 11 excluded due to failure to measure central systolic BP, arrhythmia, severe valvular disease, heart failure defined as left ventricular EF <50%, left/right arm BP difference >5mmHg |
| Bui et al, 2022 [15] | 59 | 62 | ±10 | 81.4 | Oscillometric, BP+ device | Fluid-filled | Catheter pulled back | Simultaneous with invasive brachial BP | Left/right arm BP difference >5mmHg |
| Bui et al, 2022 [15] | 179 | 61 | ±10 | 68.7 | Oscillometric,  SphygmoCor Xcel device | Fluid-filled | Catheter pulled back | Simultaneous with invasive brachial BP | 85 excluded due to left/right arm BP difference >5mmHg and right arms. |
| Pucci et al, unpublished | 29 | 68 | ±11 | 86.2 | Oscillometric,  Omron HEM9000AI | Fluid-filled | Catheter pulled back | Simultaneous with invasive brachial BP | History of peripheral arterial disease, aortic aneurysm, absent brachial or radial pulses or known obstructive large artery atherosclerotic disease, active malignancy, hypotension (<90 mm Hg), valvular heart disease, known left ventricular dysfunction (ejection fraction <50%) or arrhythmias (including frequent ventricular and supraventricular premature beats), left/right arm BP difference >5mmHg |
| Kowalski et al, 2021 [13] | 303 | 66 | ±10 | 73.6 | Oscillometric, Mobil-O-Graph device | Fluid-filled | Catheter pulled back | Sequential cuff BP first then invasive brachial BP | Failure to measure non-invasive and intraarterial BP due to technical, safety or time constraints, or consent was withdrawn; inter-arm SBP difference > 5mmHg, femoral access |
| *n (samples) included for this analysis, samples different from their published data due to additional cases collected afterwards (Cheng et al, 2010 [11]) and due to exclusion of cases with negative amplification equal or greater than 5 mmHg (Bui et al, 2022 [15])  †Data are mean ± SD | | | | | | | | | |

| Supplementary Table 2. Cuff and invasive blood pressure measurements stratified by BP devices (studies), n=795 | | | | | | | | | | | | | | |
| --- | --- | --- | --- | --- | --- | --- | --- | --- | --- | --- | --- | --- | --- | --- |
|  | Cheng et al, 2010  (n=115) | | Lin et al, 2012  (n=77) | | Ding et al, 2013  (n=33) | | Bui et al, 2022  (n=59) | | Bui et al, 2022  (n=179) | | Pucci et al  (n=29) | | Kowalski et al, 2021  (n=303) | |
| Cuff BP (mmHg) |  |  |  |  |  |  |  |  |  |  |  |  |  |  |
| SBP | 137 | ±19 | 136 | ±20 | 137 | ±19 | 133 | ±17 | 131 | ±18 | 138 | ±17 | 123 | ±16 |
| DBP | 77 | ±12 | 77 | ±11 | 77 | ±11 | 83 | ±11 | 75 | ±10 | 68 | ±11 | 76 | ±10 |
| PP | 60 | ±13 | 58 | ±17 | 60 | ±14 | 50 | ±10 | 56 | ±14 | 70 | ±15 | 47 | ±11 |
| Invasive brachial BP (mmHg) |  |  |  |  |  |  |  |  |  |  |  |  |  |  |
| SBP | 143 | ±21 | 140 | ±20 | 156 | ±25 | 136 | ±21 | 138 | ±21 | 149 | ±19 | 132 | ±21 |
| DBP | 72 | ±11 | 70 | ±11 | 78 | ±11 | 66 | ±9 | 67 | ±9 | 67 | ±10 | 69 | ±10 |
| PP | 72 | ±18 | 70 | ±20 | 78 | ±20 | 70 | ±17 | 71 | ±18 | 81 | ±19 | 64 | ±18 |
| Invasive aortic BP (mmHg) |  |  |  |  |  |  |  |  |  |  |  |  |  |  |
| SBP | 135 | ±21 | 135 | ±20 | 138 | ±22 | 130 | ±22 | 131 | ±22 | 141 | ±18 | 125 | ±20 |
| DBP | 70 | ±11 | 71 | ±10 | 78 | ±10 | 69 | ±9 | 69 | ±9 | 71 | ±10 | 69 | ±10 |
| Invasive BP amplification (mmHg)* |  |  |  |  |  |  |  |  |  |  |  |  |  |  |
| SBP | 8.0 | ±7.0 | 5.6 | ±5.6 | 17.9 | ±13.4 | 5.5 | ±8.7 | 7.3 | ±8.9 | 7.9 | ±10.9 | 6.7 | ±9.3 |
| DBP | 1.3 | ±4.1 | –0.3 | ±2.6 | 0.1 | ±6.7 | –2.9 | ±3.7 | –2.1 | ±3.4 | –3.9 | ±3.9 | –0.3 | ±4.7 |
| PP | 6.7 | ±6.6 | 5.8 | ±6.4 | 17.8 | ±9.3 | 8.4 | ±6.9 | 9.4 | ±8.5 | 11.8 | ±10.5 | 7.0 | ±9.1 |
| Cuff SBP corrected for SBP amplification (mmHg)† | 145 | ±20 | 141 | ±22 | 155 | ±26 | 138 | ±19 | 138 | ±19 | 146 | ±19 | 130 | ±18 |
| Cuff BP differences (mmHg)‡ |  |  |  |  |  |  |  |  |  |  |  |  |  |  |
| Cuff SBP – invasive brachial SBP | –6.0 | ±9.7 | –4.3 | ±11.1 | –18.7 | ±11.8 | –3.2 | ±8.0 | –6.8 | ±10.8 | –10.5 | ±12.3 | –9.0 | ±13.2 |
| Cuff DBP – invasive brachial DBP | 5.7 | ±7.7 | 7.0 | ±10.2 | –1.3 | ±7.3 | 16.7 | ±6.8 | 7.9 | ±5.0 | 1.1 | ±6.1 | 7.5 | ±8.3 |
| Cuff PP – invasive brachial PP | –11.7 | ±10.7 | –11.3 | ±12.1 | –17.4 | ±9.4 | –19.8 | ±10.1 | –14.7 | ±10.6 | –11.6 | ±13.4 | –16.5 | ±14.4 |
| Data are mean ± standard deviation; BP: blood pressure; SBP: systolic blood pressure; DBP: diastolic blood pressure; PP: pulse pressure;  *Invasive BP amplification was defined as invasive brachial BP minus invasive aortic BP;  †Individual cuff SBP was added by each corresponding SBP amplification;  ‡Cuff BP differences were defined as cuff BP minus invasive brachial BP | | | | | | | | | | | | | | |

| Supplementary Table 3. Participant characteristics, cuff, clinical and invasive blood pressure measurements stratified by excluded and complete data | | | | | |
| --- | --- | --- | --- | --- | --- |
| Variables | n=40  (Missing height and heart rate) | | n=755  (Complete data) | | p values |
| Participant characteristics |  | |  | |  |
| Male sex, n (%) | 33 | (82.5) | 552 | (73.1) | 0.189 |
| Age (years) | 67 | ±10 | 63 | ±11 | 0.065 |
| Coronary artery disease, n (%)† | 7 | (63.6) | 401 | (53.1) | 0.487 |
| Cuff BP (mmHg) |  |  |  |  |  |
| SBP | 136 | ±17 | 130 | ±19 | 0.027 |
| DBP | 72 | ±13 | 77 | ±11 | 0.005 |
| PP | 65 | ±16 | 53 | ±14 | <0.001 |
| MAP | 93 | ±12 | 94 | ±12 | 0.580 |
| Heart rate (bpm) | 68 | ±12 | 67 | ±12 | 0.556 |
| Invasive brachial BP (mmHg) |  |  |  |  |  |
| SBP | 145 | ±20 | 137 | ±22 | 0.025 |
| DBP | 67 | ±10 | 69 | ±10 | 0.285 |
| PP | 78 | ±19 | 68 | ±19 | 0.001 |
| MAP | 93 | ±11 | 92 | ±12 | 0.477 |
| Invasive aortic BP (mmHg) |  |  |  |  |  |
| SBP | 138 | ±20 | 130 | ±21 | 0.018 |
| DBP | 71 | ±11 | 70 | ±10 | 0.522 |
| MAP | 93 | ±12 | 90 | ±12 | 0.080 |
| Invasive BP amplification (mmHg)‡ |  |  |  |  |  |
| SBP | 7.1 | ±9.9 | 7.4 | ±9.1 | 0.840 |
| DBP | -3.5 | ±3.9 | –0.6 | ±4.4 | <0.001 |
| PP | 10.6 | ±9.4 | 8.0 | ±8.6 | 0.065 |
| Cuff SBP corrected with SBP amplification (mmHg)§ | 143 | ±18 | 137 | ±21 | 0.058 |
| Cuff BP differences (mmHg)\|\| |  |  |  |  |  |
| Cuff SBP – invasive brachial SBP | -8.8 | ±12.3 | –7.6 | ±11.9 | 0.528 |
| Cuff DBP – invasive brachial DBP | 4.4 | ±7.9 | 7.5 | ±8.3 | 0.019 |
| Cuff PP – invasive brachial PP | -13.2 | ±12.4 | –15.1 | ±12.6 | 0.342 |
| Data are mean ± standard deviation; BP: blood pressure; SBP: systolic blood pressure; DBP: diastolic blood pressure; PP: pulse pressure; MAP: mean arterial pressure  *Hypertension was based on cuff, invasive brachial and aortic BP values and defined as SBP≥130mmHg and/or DBP≥80mmHg according to the 2017 ACC/AHA guidelines;  †n=11/40;  ‡Invasive BP amplification was defined as invasive brachial BP minus invasive aortic BP;  §Individual cuff SBP was added by each corresponding SBP amplification;  \|\|Cuff BP differences were defined as cuff minus invasive brachial BP | | | | | |

| Supplementary Table 4. Association between cuff SBP, DBP and PP accuracy and invasive SBP amplification | | | | | | |
| --- | --- | --- | --- | --- | --- | --- |
|  | n* | β† | 95%CI | | R^2^(%)‡ | p |
| Cuff – invasive brachial SBP |  |  |  |  |  |  |
| Unadjusted | 742 | –0.57 | (–0.65 | ;–0.48) | 18.5 | <0.001 |
| Adjusted§ | 742 | –0.52 | (–0.60 | ;–0.44) | 33.0 | <0.001 |
| Cuff – invasive brachial DBP |  |  |  |  |  |  |
| Unadjusted | 742 | –0.11 | (–0.18 | ;–0.05) | 1.40 | 0.001 |
| Adjusted§ | 742 | –0.08 | (–0.14 | ;–0.01) | 11.2 | 0.023 |
| Cuff – invasive brachial PP |  |  |  |  |  |  |
| Unadjusted | 742 | –0.45 | (–0.55 | ;–0.36) | 10.6 | <0.001 |
| Adjusted§ | 742 | –0.44 | (–0.53 | ;–0.35) | 25.2 | <0.001 |
| SBP: systolic blood pressure; DBP: diastolic blood pressure; PP: pulse pressure  *: due to missing data, complete case analysis was conducted;  † unstandardised beta, invasive SBP amplification was defined as invasive brachial SBP minus invasive aortic SBP;  ‡ adjusted R^2^;  § adjusted for sex, age, height, coronary artery disease, hypertensive medication use, heart rate, and mean invasive brachial arterial pressure | | | | | | |

| Supplementary Table 5. Participants clinical characteristics and blood pressure measures according to SBP amplification quintiles (n=795) | | | | | | | | | | | |
| --- | --- | --- | --- | --- | --- | --- | --- | --- | --- | --- | --- |
|  | 1^st^ quintile | | 2^nd^ quintile | | 3^rd^ quintile | | 4^th^ quintile | | 5^th^ quintile | | p values |
| Participant characteristics |  |  |  |  |  |  |  |  |  |  |  |
| Male sex, n (%) | 109 | (69.4) | 111 | (69.4) | 118 | (73.8) | 125 | (78.6) | 122 | (76.7) | 0.220 |
| Age (years) | 65 | ±10 | 65 | ±10 | 63 | ±12 | 63 | ±11 | 62 | ±11 | 0.019 |
| Height (cm) | 167.0 | ±10.3 | 167.3 | ±9.6 | 168.3 | ±9.7 | 169.6 | ±10.1 | 170.3 | ±9.2 | 0.013 |
| Weight (kg) | 80.8 | ±18.8 | 79.0 | ±16.6 | 79.8 | ±18.1 | 79.7 | ±17.6 | 83.4 | ±18.7 | 0.231 |
| Body mass index (kg/m^2^) | 28.8 | ±5.6 | 28.1 | ±4.7 | 27.9 | ±4.9 | 27.6 | ±4.7 | 28.6 | ±5.1 | 0.213 |
| eGFR (ml/min/1.73m^2^) | 80.0 | ±18.9 | 79.2 | ±19.1 | 80.0 | ±19.2 | 79.4 | ±19.6 | 79.5 | ±20.7 | 0.995 |
| Coronary artery disease, n (%) | 88 | (58.3) | 60 | (39.5) | 88 | (56.4) | 84 | (54.9) | 88 | (57.1) | 0.005 |
| Type 2 diabetes mellitus, n (%) | 42 | (27.8) | 33 | (21.7) | 43 | (27.7) | 35 | (23.0) | 53 | (34.4) | 0.100 |
| Cuff BP (mmHg) |  |  |  |  |  |  |  |  |  |  |  |
| SBP | 131 | ±18 | 131 | ±18 | 131 | ±18 | 127 | ±18 | 131 | ±20 | 0.327 |
| DBP | 78 | ±11 | 76 | ±11 | 77 | ±11 | 75 | ±10 | 77 | ±12 | 0.270 |
| PP | 53 | ±14 | 55 | ±14 | 54 | ±13 | 52 | ±14 | 54 | ±14 | 0.606 |
| MAP | 95 | ±12 | 94 | ±12 | 95 | ±12 | 92 | ±11 | 95 | ±13 | 0.241 |
| Heart rate (bpm) | 66 | ±11 | 64 | ±11 | 66 | ±12 | 67 | ±12 | 70 | ±13 | 0.001 |
| Invasive brachial BP (mmHg) |  |  |  |  |  |  |  |  |  |  |  |
| SBP | 133 | ±22 | 135 | ±21 | 138 | ±20 | 136 | ±21 | 146 | ±23 | <0.001 |
| DBP | 68 | ±11 | 69 | ±10 | 70 | ±10 | 68 | ±10 | 70 | ±11 | 0.220 |
| PP | 65 | ±19 | 66 | ±19 | 68 | ±17 | 68 | ±18 | 76 | ±19 | <0.001 |
| MAP | 90 | ±13 | 91 | ±11 | 93 | ±12 | 91 | ±12 | 95 | ±13 | 0.001 |
| Invasive aortic BP (mmHg) |  |  |  |  |  |  |  |  |  |  |  |
| SBP | 137 | ±22 | 133 | ±21 | 132 | ±20 | 125 | ±21 | 125 | ±21 | <0.001 |
| DBP | 71 | ±11 | 70 | ±9 | 71 | ±11 | 68 | ±10 | 69 | ±10 | 0.023 |
| MAP | 93 | ±13 | 91 | ±11 | 91 | ±12 | 87 | ±11 | 88 | ±12 | <0.001 |
| SBP amplification (mmHg) | –3.9 | ±4.8 | 2.3 | ±1.2 | 6.3 | ±1.2 | 10.9 | ±1.7 | 20.9 | ±6.3 | <0.001 |
| Data are mean ± standard deviation or n (%); BP: systolic blood pressure, SBP: systolic blood pressure; DBP: diastolic blood pressure; MAP: mean arterial pressure; eGFR: Estimated glomerular filtration rate; | | | | | | | | | | | |

| Supplementary Table 6. Concordance of BP classification according to cuff and invasive brachial BP* | | | | | | | | |
| --- | --- | --- | --- | --- | --- | --- | --- | --- |
| Invasive brachial BP classification | Brachial cuff BP classification | | | | | | | |
|  | Normal | | High-normal | | Stage 1 | | Stage 2 | |
|  | n | (%) | n | (%) | n | (%) | n | (%) |
|  | Standard brachial cuff BP | | | | | | | |
| Normal BP | ***259*** | ***(65.9)*** | 27 | (15.8) | 8 | (4.9) | 1 | (1.5) |
| High-normal BP | 85 | (21.6) | ***60*** | ***(35.1)*** | 15 | (9.2) | 0 |  |
| Stage 1 Hypertension | 48 | (12.2) | 71 | (41.5) | ***96*** | ***(58.5)*** | 15 | (22.4) |
| Stage 2 Hypertension | 1 | (0.3) | 13 | (7.6) | 45 | (27.4) | ***51*** | ***(76.1)*** |
|  | (%) Agreement: 58.6%, kappa: 0.41, p<0.001 | | | | | | | |
|  | Cuff SBP corrected with SBP amplification† | | | | | | | |
| Normal BP | ***226*** | ***(80.7)*** | 48 | (27.1) | 18 | (7.8) | 3 | (2.8) |
| High-normal BP | 41 | (14.6) | ***71*** | ***(40.1)*** | 46 | (19.8) | 2 | (1.9) |
| Stage 1 Hypertension | 12 | (4.3) | 57 | (32.2) | ***132*** | ***(56.9)*** | 29 | (27.4) |
| Stage 2 Hypertension | 1 | (0.4) | 1 | (0.6) | 36 | (15.5) | ***72*** | ***(67.9)*** |
|  | (%) Agreement: 63.0%, kappa: 0.49, p<0.001 | | | | | | | |
| BP: systolic blood pressure, SBP: systolic blood pressure; DBP: diastolic blood pressure  *Hypertension classification based on 2020 ISH guidelines: Normal BP: SBP <130 mmHg and DBP <85 mmHg; High-normal BP: SBP 130-139 mmHg and/or DBP 85-89 mmHg; Stage 1 Hypertension: SBP 140-159 mmHg and/or DBP 90-99 mmHg; Stage 2 Hypertension: SBP ≥160 mmHg and/or DBP ≥100 mmHg;  Each column adds to 100%;  n (%) represent the number and percentage of concordance for each classification, bolded and italic numbers represent concordant classifications;  †Individual SBP amplification value was added to each corresponding cuff SBP value before applying ISH classification | | | | | | | | |

| Supplementary Table 7. Concordance of BP classification according to cuff and invasive brachial BP* | | | | | | | | | | | | | |
| --- | --- | --- | --- | --- | --- | --- | --- | --- | --- | --- | --- | --- | --- |
|  | Brachial cuff BP classification | | | | | | | | | | | | |
| Invasive brachial BP classification | Optimal | | Normal | | | High-normal | | Stage 1 | | Stage 2 | | Stage 3 | |
|  | n | (%) | n | | (%) | n | (%) | n | (%) | n | (%) | n | (%) |
| Standard brachial cuff BP | | | | | | | | | | | | | |
| Optimal BP | ***125*** | ***(55.1)*** | 24 | | (14.5) | 4 | (2.3) | 2 | (1.2) | 0 |  | 0 |  |
| Normal BP | 60 | (26.4) | ***50*** | | ***(30.1)*** | 23 | (13.5) | 6 | (3.7) | 1 | (1.7) | 0 |  |
| High-normal BP | 27 | (11.9) | 58 | | (34.9) | ***60*** | ***(35.1)*** | 15 | (9.2) | 0 |  | 0 |  |
| Stage 1 | 14 | (6.2) | 34 | | (20.5) | 71 | (41.5) | ***96*** | ***(58.5)*** | 15 | (25.4) | 0 |  |
| Stage 2 | 1 | (0.4) | 0 | |  | 13 | (7.6) | 40 | (24.4) | ***22*** | ***(37.3)*** | 2 | (25.0) |
| Stage 3 | 0 |  | 0 | |  | 0 |  | 5 | (3.1) | 21 | (35.6) | ***6*** | ***(75.0)*** |
|  | | | | (%) Agreement: 45.2%, kappa: 0.31, p<0.001 | | | | | | | | | |
| Cuff SBP corrected with SBP amplification† | | | | | | | | | | | | | |
| Optimal BP | ***101*** | ***(74.3)*** | 41 | | (28.5) | 11 | (6.2) | 2 | (0.9) | 0 |  | 0 |  |
| Normal BP | 22 | (16.2) | ***62*** | | ***(43.1)*** | 37 | (20.9) | 16 | (6.9) | 3 | (3.8) | 0 |  |
| High-normal BP | 10 | (7.4) | 31 | | (21.5) | ***71*** | ***(40.1)*** | 46 | (19.8) | 2 | (2.5) | 0 |  |
| Stage 1 | 2 | (1.5) | 10 | | (6.9) | 57 | (32.2) | ***132*** | ***(56.9)*** | 29 | (36.7) | 0 |  |
| Stage 2 | 1 | (0.7) | 0 | |  | 1 | (0.6) | 34 | (14.7) | ***34*** | ***(43.0)*** | 8 | (29.6) |
| Stage 3 | 0 |  | 0 | |  | 0 |  | 2 | (0.9) | 11 | (13.9) | ***19*** | ***(70.4)*** |
|  | | | | (%) Agreement: 52.7%, kappa: 0.40, p<0.001 | | | | | | | | | |
| BP: systolic blood pressure, SBP: systolic blood pressure; DBP: diastolic blood pressure  *Hypertension classification based on 2018 ESH guidelines: Optimal BP: SBP <120 mmHg and DBP <80 mmHg; Normal BP: SBP 120-129 mmHg and/or DBP 80-84 mmHg; High-normal BP: SBP 130-139 mmHg and/or DBP 85-89 mmHg; Stage 1 Hypertension: SBP 140-159 mmHg and/or DBP 90-99 mmHg; Stage 2 Hypertension: SBP 160-179 mmHg and/or DBP 100-109 mmHg; Stage 3 Hypertension: SBP ≥180 mmHg and/or DBP ≥110 mmHg;  Each column adds to 100%;  n (%) represent the number and percentage of concordance for each classification, bolded and italic numbers represent concordant classifications;  †Individual SBP amplification value was added to each corresponding cuff SBP value before applying ESH classification | | | | | | | | | | | | | |
